# A Novel Ferroptosis-Related Long Non-Coding RNA Prognostic Signature Correlates With Genomic Heterogeneity, Immunosuppressive Phenotype, and Drug Sensitivity in Hepatocellular Carcinoma

**DOI:** 10.3389/fimmu.2022.929089

**Published:** 2022-07-08

**Authors:** Guanghao Li, Yongheng Liu, Yanting Zhang, Yao Xu, Jin Zhang, Xianfu Wei, Zhongmin Zhang, Chao Zhang, Jinyan Feng, Qiang Li, Guowen Wang

**Affiliations:** ^1^ Department of Bone and Soft Tissue Tumors, Tianjin Medical University Cancer Institute and Hospital, National Clinical Research Center for Cancer, Key Laboratory of Cancer Prevention and Therapy, Tianjin’s Clinical Research Center for Cancer, Tianjin, China; ^2^ Department of Hepatobiliary Cancer, Tianjin Medical University Cancer Institute and Hospital, National Clinical Research Center for Cancer, Key Laboratory of Cancer Prevention and Therapy, Tianjin’s Clinical Research Center for Cancer, Tianjin, China

**Keywords:** ferroptosis, HCC, prognosis, tumor heterogeneity, tumor microenvironment

## Abstract

Hepatocellular carcinoma (HCC) with high heterogeneity is a common malignancy worldwide, but effective treatments are limited. Ferroptosis plays a critical role in tumors as a novel iron-dependent and reactive oxygen species-reliant type of cell death. Several studies have shown that long non-coding RNAs (lncRNAs) can drive HCC initiation and progression. However, the prognostic value of ferroptosis-related lncRNAs in patients with HCC has not been explored comprehensively. Gene set variation analysis (GSVA) based on gene set and RNA-seq profiles obtained from public databases indicated that ferroptosis is suppressed in HCC patients. Ferroptosis-related differentially expressed lncRNAs were screened by Pearson’s test. Univariate Cox regression, least absolute shrinkage and selection operator (LASSO) regression, and multivariate Cox regression were performed to establish a novel five ferroptosis-related lncRNA signature in the training cohort with 60% patients, which was further verified in the testing cohort with 40% patients. Dimensionality reduction analysis, Kaplan–Meier curve, receiver operating characteristic (ROC) curve, independent prognostic analysis, and stratification analysis confirmed that our signature had a high clinical application value in predicting the overall survival of HCC patients. Compared to the clinicopathological factors and the other four published HCC prognostic signatures, the current risk model had a better predictive value. The comparison results of functional enrichment, tumor immune microenvironment, genomic heterogeneity, and drug sensitivity between the high- and low-risk groups showed that the risk score is associated with extensive genomic alterations, immunosuppressive tumor microenvironment, and clinical treatment response. Finally, cell experiments showed that silencing LNCSRLR expression inhibited the growth, proliferation, migration, and invasion of the HCC cell line. Thus, the model can function as an efficient indicator for predicting clinical prognosis and treatment of anticancer drugs in HCC patients.

## Introduction

Hepatocellular carcinoma (HCC) is the sixth most common and fourth-most fatal malignancy worldwide, accounting for 75%–85% of primary liver cancers, with 1- and 3-year survival rates of 20% and 5%, respectively, and a median survival of only 8 months (1, 2). The underlying pathogenic elements for HCC include infections due to aflatoxin exposure, hepatitis virus, alcohol abuse, type 2 diabetes, and obesity (3, 4). Although alpha-fetoprotein (AFP) has been widely used for early diagnosis and surveillance of HCC, its high false-positive rate missed AFP-negative HCC diagnosis frequently (5). Various clinical strategies for HCC treatment can include surgical resection, liver transplantation, radiotherapy, and systemic chemotherapy; however, the therapeutic outcome is limited due to the high recurrence rate (6). Therefore, developing an effective therapeutic target for HCC is an urgent requirement.

Ferroptosis is a form of iron-dependent regulated cell death, mediated by lipid accumulation of reactive oxygen species (ROS) and characterized by cell shrinkage and increased mitochondrial membrane concentration (7, 8). Although the specific role and regulatory mechanisms of ferroptosis in tumors are unclear, studies have reported that it is involved in various cancers, including breast cancer (9), ovarian cancer (10), pancreatic cancer (11), and HCC (12). A growing body of evidence suggests that ferroptosis inhibition plays a major role in all types of cancer treatment responses. In HCC, the polymorphism of *TP53* (the S47 variant) and *CISD1* (S47 variant) genes have a negative regulatory effect on ferroptosis, suggesting a critical role for the related genes in tumor progression (13, 14). In addition, several bioinformatics studies showed that ferroptosis-related gene signatures could predict the survival outcomes of HCC patients (15, 16). The key role of ferroptosis in tumors deems it a potential target for anticancer treatment.

Long non-coding RNAs (lncRNAs) are a class of non-coding transcripts with a minimum length of 200 nucleotides (17). Dynamic changes in the expression and mutation of lncRNAs are closely related to tumorigenesis, tumor progression, metastasis, and cancer immunity. Thus, lncRNAs are emerging as novel biomarkers and therapeutic targets for novel strategies in cancer therapy (18–20). For example, the expression of lncRNA HULC has a close correlation with the tumor size and the overall survival (OS) of HCC. It drives the development of HCC hallmarks, such as proliferation, migration, and invasion, by regulating the phosphorylation of YB-1 (21). Several ferroptosis-related lncRNA signatures have been reported in HCC; however, none of them had systematically explored the correlation between the lncRNA signature and tumor heterogeneity (22, 23). Therefore, it is crucial to systematically elucidate the underlying function of ferroptosis-related lncRNAs in HCC.

Here, we mined the data from public databases to identify the difference in ferroptosis-related processes between normal and tumor samples and to construct a ferroptosis-related lncRNA signature for HCC. Furthermore, the correlation between the signature and clinical outcome, genomic heterogeneity, cancer markers, tumor immune microenvironment, and clinical treatment response was assessed to explore the underlying mechanisms. Thus, the current study might be valuable for prognosis prediction and provide new perspectives for the selection of clinical treatment for HCC.

## Materials and Methods

### Data and Resources

The clinical and RNA-seq data (fragments per kilobase of transcript per million mapped reads (FPKM) format) of HCC were obtained from The Cancer Genome Atlas (TCGA) portal (http://tcga-data.nci.nih.gov/) and International Cancer Genome Consortium (ICGC) database (https://dcc.icgc.org/). A total of 259 ferroptosis-associated genes (driver, 108; suppressor, 69; marker, 111) were obtained from the FerrDB platform (http://www.zhounan.org/ferrdb/) (**Supplementary Table 1**).

### Gene Set Variation Analysis

The RNA-seq data of paired tumor-normal samples from TCGA and ICGC cohorts were extracted according to the sample name and type. The ferroptosis activity score was calculated using the gene set variation analysis (GSVA) method in the R package. Three ferroptosis gene sets were obtained from FerrDB to calculate the paired HCC samples from TCGA and ICGC cohorts. A false discovery rate (FDR) <0.05 is considered significant dysregulation.

### Identification of Differentially Expressed Ferroptosis-Associated Long Non-Coding RNAs

A total of 14,142 lncRNAs were extracted from TCGA HCC dataset according to the annotation file acquired from the GENCODE website (https://www.gencodegenes.org/). Pearson’s analysis examined the correlation between ferroptosis-related genes and corresponding lncRNAs. Then, 1,271 ferroptosis-related lncRNAs with the threshold value |cor| > 0.4, *p* < 0.001, were identified. “Limma” R package was applied to identify the differentially expressed lncRNAs between 374 HCC samples and 50 adjacent normal liver tissue samples with the thresholds of |log2 fold-change| > 1 and FDR < 0.05. Subsequently, 781 differentially expressed ferroptosis-related lncRNAs were identified.

### Construction and Validation of Ferroptosis-Related Long Non-Coding RNA Signature

TCGA HCC patients were randomly assigned to the training and testing cohorts in a 6:4 ratio. The training cohort was used to construct a ferroptosis-associated lncRNA model, and the testing cohort was utilized to validate this model. Univariate Cox regression analysis was performed to sort the prognostic lncRNAs related to the OS (*p* < 0.05) in the training cohort. Least absolute shrinkage and selection operator (LASSO) penalized Cox regression analysis was conducted on these prognostic lncRNAs using the R “glmnet” package. Optimal penalty parameter (lambda) was selected by the minimum 10-fold cross-validation. The candidate lncRNAs were incorporated into multivariate Cox regression analysis to evaluate their contribution as prognostic factors in the OS of patients. Finally, a prognostic model containing five ferroptosis-related lncRNAs was constructed based on the lowest Akaike information criterion (AIC) value. The computational formula of the risk score for each patient is as follows: Risk score = Coefficient lncRNA1 × expression of lncRNA1) + (Coefficient lncRNA2 × expression of lncRNA2) + ⋯ + (Coefficient lncRNAn × expression lncRNAn). TCGA HCC patients were categorized into the high- and low-risk groups based on the median risk score. Principal component analysis (PCA), t-distributed stochastic neighbor embedding (t-SNE), and Uniform Manifold Approximation and Projection (UMAP) were performed using “stats,” “Rtsne,” and “umap” packages for dimensionality reduction analysis and to examine the clustering ability of the signature. The Kaplan–Meier curve was used to calculate the survival difference between the high- and low-risk groups using the “survival” and “survminer” R packages. The clinicopathological parameters, such as age, gender, grade, and stage, were included in the univariate and multivariate Cox proportional hazards regression models to elucidate whether the risk score is an independent prognostic indicator. The receiver operating characteristic (ROC) curve was generated using “timeROC” R package to assess the predictive power of the signature. Stratification analysis evaluated the correlation between the risk score and clinical characteristics. In addition, a prognostic nomogram was created using “regplot” R packages based on the clinicopathological parameters and risk score to predict the 1-, 3-, and 5-year OS probability of HCC patients. Finally, the ROC and calibration curves were used to assess the performance of the nomograms.

### Tumor Genomic Heterogeneity Analysis

Copy number variation (CNV) data obtained from the UCSC Xena browser (http://xena.ucsc.edu) were used to calculate the copy number alteration (CNA) burden using the CNApp tool (https://tools.idibaps.org/CNApp/). The somatic mutation data were obtained from TCGA portal for gene mutation analysis, and the quantity and quality of gene mutations were visualized using the “maftools” R package. Survival analyses of the TP53 mutation were utilized from the CVCDAP database (https://omics.bjcancer.org/cvcdap/home.do).

### Correlation Analysis of Risk Score and Cancer Markers

Stem cell indices based on the transcriptome of each TCGA HCC sample were downloaded from the UCSC Xena browser and referred to as RNAss in the following sections. The InferHeterogeneity function of the Maftools package was used to calculate the Mutant-Allele Tumor Heterogeneity (MATH) score. Neoantigen, ploidy, homologous recombination deficiency (HRD), and loss of heterozygosity (LOH) data were obtained from a previous study (24). These data and the current risk score were integrated for further analysis between the high- and low-risk groups.

### Estimation of the Immune Landscape Between Different Risk Groups

TCGA immune subtype data obtained from UCSC Xena Browser were used to investigate the correlation between the risk score and six immune subtypes. The correlation between the risk score and tumor immune microenvironment was quantified by the ESTIMATE algorithm (https://cran.r-project.org/mirrors.html), including immune score, stromal score, and ESTIMATE score (the sum of the previous two data points). To investigate the correlations between risk scores and tumor immunoregulation-related genes, a list of immunosuppressive checkpoints from the TISIDB web portal (http://cis.hku.hk/TISIDB/index.php) was compiled. The cancer immune cycle is a process wherein the immune system recognizes and kills the cancer cells; thus, the relative activity of the seven steps of the cancer-immune cycle between the high- and low-risk groups was evaluated using the TIP pipeline (http://biocc.hrbmu.edu.cn/TIP/).

### Drug Sensitivity Analysis

In order to predict the chemotherapeutic response in the high- and low-risk groups, half-maximal inhibitory concentration (IC50) of various chemotherapy drugs recommended for HCC treatment were calculated *via* the “pRRophetic” R package. In addition, two currently well-known algorithms were used to assess the clinical response of immune therapy in different risk groups, including TIDE (Tumor Immune Dysfunction and Exclusion) (http://tide.dfci.harvard.edu) and ImmuCellAI (Immune Cell Abundance Identifier) (http://bioinfo.life.hust.edu.cn/ImmuCellAI#!/).

### Functional Enrichment Analysis

In order to identify the potential molecular mechanisms or potential functional pathways that involve the ferroptosis-related lncRNA signature, gene set enrichment analysis (GSEA) was performed using R package ClusterProfiler, and the results were visualized using R package ggplot2. FDR < 0.25 and p.adjust < 0.05 were considered statistically significant.

### Cell Culture and Quantitative Real-Time PCR

The human immortalized normal liver cell line LO2 and HCC cells Hep3B were cultured in Dulbecco’s modified Eagle’s medium (DMEM; Gibco, Grand Island, NY, USA) containing 1% penicillin/streptomycin (Gibco) and 10% fetal bovine serum (FBS) (Gibco). The cells were placed in a humidified incubator maintained at 37°C with 5% CO_2_. Total RNA from cells was isolated using TRIzol Reagent (Invitrogen, Carlsbad, CA, USA) according to the manufacturer’s protocol. The cDNA synthesis was reverse-transcribed using the Transcriptor First Strand cDNA Synthesis Kit (GenStar, Beijing, China). The RT-qPCR assay was conducted with LightCycler 480 Fluorescence Quantitative System (Roche, Basel, Switzerland). The relative gene expression levels of LNCSRLR were calculated by the 2^−ΔΔCt^ method, and each sample was tested in triplicate. The sequences of all primers used in this study are provided in **Supplementary Table 2**.

### Plasmid Construction and Transfection

Plasmids and RNA interference sequences si-LNCSRLR (5′-GUUACUGUACAUCAGGAAUTT-3′) and si-NC (5′-UUCUCCGAACGUGUCACGUTT-3′) were synthesized by GENECHEM (Shanghai, China). The cells were seeded in the culture plate 1 day before transfection, and transfection was carried out when the cell confluence reached 70%. According to the instructions provided by the reagent manufacturer, the targeted cells were transfected. The cells were incubated in a CO_2_ incubator at 37°C after transfection. After 6 h of incubation, the culture medium containing serum was replaced by medium + 10% FBS. The transfection efficiency was measured by RT-qPCR 48 h after transfection.

### Cell Counting Kit-8 Assay

In 96-well plates, 2 × 10^3^ cells were seeded and incubated with 5 duplicates. Following the manufacturer’s instructions, 10 μl of Cell Counting Kit-8 (CCK-8) (MCE, NJ, USA) was added per plate well. After 2 h in the 37°C incubators, the plate was measured at 450 nm (CCK-8).

### Clone Formation Assay

In 6-well plates, 200 cells were seeded after being transfected for 48 h. The cells were re-transfected on day 6, and the culture medium was changed every 2 days. After 10 days, cells were fixed with 4% paraformaldehyde (Solarbio, Beijing, China) and dyed with 0.5% crystal violet (Solarbio, Beijing, China) for 10 min. Cell colonies of more than 50 cells were counted by ImageJ.

### Scratch Assay

A total of 7 × 10^5^ cells were seeded in each well of a 6-well plate for 24 h. A line was drawn in the middle of the cells with a 10-μl pipette tip. After being washed twice with phosphate-buffered saline (PBS), cells were cultured in Roswell Park Memorial Institute (RPMI)-1640 with 1% FBS for 48 h in a 37°C incubator, and wounds were photographed by a microscope at different time intervals. The distance of the wounds was measured by Photoshop.

### Migration and Invasion Assay

The migration and invasion capacities of si-control and si-LNCSRLR cells were analyzed by polycarbonate membranes (8-μm pore) in 24-well transwell chambers (Corning, NY, USA); 1 × 10^4^ cells in a serum-free medium containing 0.1% bovine serum albumin (BSA) were added to the upper chamber. Additional medium supplemented of 0.1% BSA and epidermal growth factor (EGF) (50 ng/ml, MCE, NJ, USA) were added into the lower chamber. After 24 h of incubation, cells in the upper chamber were completely scraped and transferred to the lower membrane. The polycarbonate membranes were fixed and stained with Giemsa solution (Solarbio, Beijing, China) and photographed by a microscope.

For invasion assay, transwell chambers were coated with prediluted extracellular matrix (3 mg/ml, Merck, Darmstadt, Germany) for 1 h before being added with cells to the upper chamber. The following steps were the same with migration.

### Statistical Analysis

All statistical analyses were performed by R language (version 4.0.4). For differences between groups, normally distributed and non-normally distributed data were assessed using the T-test and the Wilcoxon rank-sum test, respectively. If not specified above, a two-sided *p*-value <0.05 was regarded as statistically significant (*p < 0.05; **p < 0.01; ***p < 0.001; ****p < 0.0001).

## Results

### Ferroptosis Processes Are Significantly Dysregulated in Hepatocellular Carcinoma

**Figure 1:**
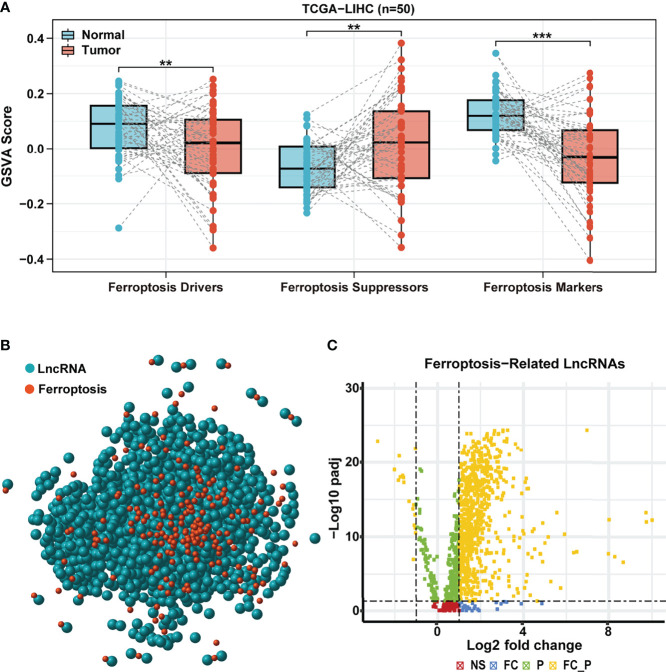
Identification of differentially expressed ferroptosis-related lncRNA. **(A)** GSVA scores of ferroptosis drive, ferroptosis suppressor, and ferroptosis maker in paired samples of TCGA HCC cohort. **(B)** Correlation network of ferroptosis-related genes (red) and corresponding lncRNAs (blue). **(C)** The volcano plot for differentially expressed ferroptosis-related lncRNAs. Yellow dot represents significantly differentially expressed lncRNAs (|log2 fold-change| > 1 and FDR < 0.05). LncRNA, long non-coding RNA; GSVA, gene set variation analysis; TCGA, The Cancer Genome Atlas; HCC, hepatocellular carcinoma; FDR, false discovery rate.

In order to explore the difference in ferroptosis processes activity between HCC and normal samples, we performed GSVA based on the expression of ferroptosis-related genes obtained from FerrDB, including ferroptosis drivers, suppressors, and markers. Compared to the paired normal adjacent tissues, the GSVA scores of ferroptosis drivers and markers were lower in TCGA HCC tissues, whereas the GSVA score of ferroptosis suppressors showed an opposite trend (**Figure 1A**). Surprisingly, similar results were observed in paired samples of the ICGC cohort (**Supplementary Figure 1A**). These results confirmed that the ferroptosis process was remarkably inhibited in HCC and might play vital roles in tumorigenesis and development.

### Identification of Differentially Expressed Ferroptosis-Related Long Non-Coding RNAs

Based on the above results, we hypothesized that lncRNAs that regulate the ferroptosis-related genes might play a critical role in HCC. A total of 259 ferroptosis-related genes obtained from FerrDB were used to assess TCGA HCC cohort further. According to Pearson’s correlation analysis (|cor| > 0.4, *p* < 0.001), we identified a total of 1,271 ferroptosis-related lncRNAs and established an lncRNA-mRNA co-expression network (red nodes, ferroptosis-related genes; blue nodes, ferroptosis-related lncRNAs) (**Figure 1B**). Then, 781 ferroptosis-related lncRNAs (764 upregulated and 17 downregulated) were identified as differentially expressed between HCC and paracancerous samples (|log2 FC | >1 and FDR < 0.05, **Figure 1C**).

### Establishment of Ferroptosis-Related Long Non-Coding RNA Signature in Hepatocellular Carcinoma

**Figure 2:**
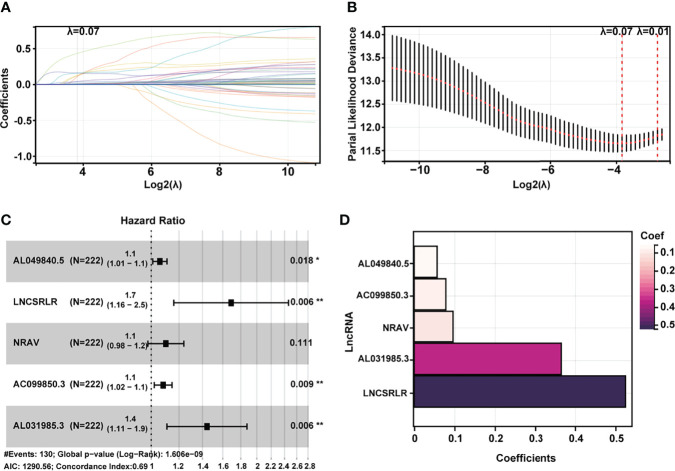
Construction of a 5-ferroptosis-related lncRNA prognostic model. **(A, B)** Screening of the candidate lncRNAs by LASSO regression analysis. **(C)** Multivariate Cox regression for the model construction. **(D)** Model coefficients of 5 critical ferroptosis-related lncRNAs. LncRNA, long non-coding RNA; LASSO, least absolute shrinkage and selection operator.

Subsequently, HCC patients were randomly divided into two independent cohorts: training (n = 222) and test (n = 148). The training cohort was used to construct a ferroptosis-associated lncRNA model, and the test cohort was applied to validate this established model. No significant differences were detected between the two groups’ baseline clinical and demographic characteristics (**Table 1**, *p* > 0.05). Fifty of 781 lncRNAs exhibited prognostic relevance by univariate Cox analysis in the training cohort (*p* < 0.05, **Table 2**). However, 50 lncRNAs showed an unfavorable prognosis in HCC patients. Based on the 50 candidate lncRNAs mentioned above, five lncRNAs (AL049840.5, NRAV, AC099850.3, LNCSRLR, and AL031985.3) were ultimately included in the prognostic signature by LASSO regression (**Figures 2A, B**) and multivariate Cox regression (**Figure 2C**). The coefficients of these lncRNAs are shown in **Figure 2D**, indicating that LNCSRLR had the highest coefficient. Then, we calculated the risk score of each patient by multiplying the expression level of each lncRNA and their corresponding multivariate Cox regression coefficients: Risk score = 0.0567813957595128 × expression of AL049840.5) + (0.0961066604661453 × expression of NRAV) + (0.0783912583024516 × expression of AC099850.3) + (0.524753638055906 × expression of LNCSRLR) + (0.365833526768369 × expression of AL031985.3).

**Table 1:** The clinical information of HCC patients obtained from the UCSC Xena browser.

Covariates	Type	Total	Test	Train	p-Value
Age	≤65	232 (62.7%)	94 (63.51%)	138 (62.16%)	0.8779
Age	>65	138 (37.3%)	54 (36.49%)	84 (37.84%)	
Gender	Female	121 (32.7%)	47 (31.76%)	74 (33.33%)	0.8387
Gender	Male	249 (67.3%)	101 (68.24%)	148 (66.67%)	
Grade	G1	55 (14.86%)	19 (12.84%)	36 (16.22%)	0.6997
Grade	G2	177 (47.84%)	70 (47.3%)	107 (48.2%)	
Grade	G3	121 (32.7%)	53 (35.81%)	68 (30.63%)	
Grade	G4	12 (3.24%)	5 (3.38%)	7 (3.15%)	
Grade	Unknown	5 (1.35%)	1 (0.68%)	4 (1.8%)	
Stage	Stage I	171 (46.22%)	70 (47.3%)	101 (45.5%)	0.7532
Stage	Stage II	85 (22.97%)	36 (24.32%)	49 (22.07%)	
Stage	Stage III	85 (22.97%)	32 (21.62%)	53 (23.87%)	
Stage	Stage IV	5 (1.35%)	3 (2.03%)	2 (0.9%)	
Stage	Unknown	24 (6.49%)	7 (4.73%)	17 (7.66%)	
T	T1	181 (48.92%)	71 (47.97%)	110 (49.55%)	0.897
T	T2	93 (25.14%)	39 (26.35%)	54 (24.32%)	
T	T3	80 (21.62%)	30 (20.27%)	50 (22.52%)	
T	T4	13 (3.51%)	6 (4.05%)	7 (3.15%)	
T	Unknown	3 (0.81%)	2 (1.35%)	1 (0.45%)	
M	M0	266 (71.89%)	109 (73.65%)	157 (70.72%)	0.39
M	M1	4 (1.08%)	3 (2.03%)	1 (0.45%)	
M	Unknown	100 (27.03%)	36 (24.32%)	64 (28.83%)	
N	N0	252 (68.11%)	107 (72.3%)	145 (65.32%)	0.8483
N	N1	4 (1.08%)	1 (0.68%)	3 (1.35%)	
N	Unknown	114 (30.81%)	40 (27.03%)	74 (33.33%)	
Fibrosis	No	74 (20%)	32 (21.62%)	42 (18.92%)	0.3059
Fibrosis	Unknown	159 (42.97%)	68 (45.95%)	91 (40.99%)	
Fibrosis	Yes	137 (37.03%)	48 (32.43%)	89 (40.09%)	
Hepatitis	No	198 (53.51%)	80 (54.05%)	118 (53.15%)	0.8105
Hepatitis	Unknown	19 (5.14%)	9 (6.08%)	10 (4.5%)	
Hepatitis	Yes	153 (41.35%)	59 (39.86%)	94 (42.34%)	
Alcoholic liver disease	No	234 (63.24%)	88 (59.46%)	146 (65.77%)	0.3347
Alcoholic liver disease	Unknown	19 (5.14%)	9 (6.08%)	10 (4.5%)	
Alcoholic liver disease	Yes	117 (31.62%)	51 (34.46%)	66 (29.73%)	

HCC, hepatocellular carcinoma.

**Table 2:** Prognostic ferroptosis-related lncRNAs identified *via* univariate cox regression analysis.

ID	HR	HR.95L	HR.95H	p-Value
PCAT6	1.101128697	1.028175925	1.179257729	0.005879471
AC026356.1	4.256425891	2.296542184	7.888886818	4.21E−06
AC004816.1	1.205912947	1.077230865	1.349966922	0.001145546
RHPN1-AS1	1.680926558	1.255261851	2.250936002	0.000490411
BACE1-AS	1.213342721	1.099544237	1.338918898	0.000118829
AP001469.3	1.98711567	1.338184204	2.950736284	0.000663853
AC124798.1	1.216415375	1.090012109	1.357476997	0.000465975
AC107959.3	1.348870014	1.138077165	1.59870558	0.000556876
AC145343.1	1.316298629	1.106654033	1.565658308	0.001903496
AC068473.5	1.412604074	1.184975876	1.68395856	0.000116626
AL049840.5	1.067995878	1.02092244	1.117239814	0.00423258
AC145207.5	1.770774959	1.36826501	2.291693444	1.41E−05
LINC01063	1.458559996	1.161804723	1.831114316	0.001145272
GSEC	1.714551783	1.327797166	2.213958495	3.57E−05
POLH-AS1	2.534038253	1.687321194	3.805647607	7.42E−06
AC016747.1	1.17507124	1.065921224	1.295398185	0.001181053
LINC00862	1.317894451	1.097523027	1.582514209	0.003109293
AL671710.1	2.694361119	1.359259158	5.340837176	0.004522701
SNHG12	1.133747835	1.036289695	1.240371453	0.006195052
LNCSRLR	2.259863708	1.597454314	3.196951508	4.09E−06
KDM4A-AS1	2.851826363	1.89761619	4.28585804	4.60E−07
AL355574.1	1.290173711	1.120599811	1.48540825	0.000394545
AC010969.2	1.321101859	1.0788301	1.617780336	0.007059071
MIR4435-2HG	1.12056819	1.040793625	1.2064573	0.002518708
CYTOR	1.03490444	1.01141968	1.058934506	0.003394852
MED8-AS1	2.44816415	1.526404	3.926553983	0.000203584
MKLN1-AS	3.377528933	2.285090306	4.992232325	1.03E−09
AP003469.2	1.325450987	1.134717732	1.548244352	0.000379007
AC009779.2	1.177292186	1.092158625	1.269061892	2.03E−05
MIR210HG	1.1407472	1.067701896	1.218789794	9.61E−05
DANCR	1.018687234	1.00467011	1.032899926	0.008817594
AC099850.1	2.249515655	1.412712565	3.581988869	0.000636297
LINC02870	1.084193323	1.032525861	1.138446217	0.001175453
NRAV	1.234701102	1.129596703	1.349585041	3.41E−06
ZFPM2-AS1	1.096086666	1.056166831	1.137515348	1.25E−06
ZEB1-AS1	1.461886231	1.179401049	1.812031077	0.000527989
FOXD2-AS1	1.154025654	1.062415656	1.253535001	0.000687105
TMCC1-AS1	2.64072479	1.846203774	3.777171033	1.05E−07
AC020915.2	1.361164093	1.112691171	1.665123025	0.0027148
AC006504.7	1.392192568	1.157676946	1.674215033	0.000438679
AL050341.2	1.140333372	1.042539071	1.247301167	0.004096727
AC074117.1	1.478011805	1.206642512	1.810411016	0.000160097
AC026401.3	1.086114688	1.038429719	1.135989364	0.000310758
SNHG3	1.068790138	1.039154618	1.099270828	3.54E−06
AC099850.3	1.144508052	1.092632084	1.198846986	1.18E−08
LINC01011	1.996524176	1.325989167	3.006139783	0.000928733
AL031985.3	1.879214586	1.546887513	2.282937466	2.10E−10
SBF2-AS1	1.434776564	1.144624975	1.798478832	0.001737623
PRRT3-AS1	1.095343996	1.041897754	1.15153187	0.000359635
LINC01138	1.399793839	1.203228954	1.628470446	1.32E−05

LncRNA, long non-coding RNA.

### Prognostic Value of the Ferroptosis-Associated Long Non-Coding RNA Signature

**Figure 3:**
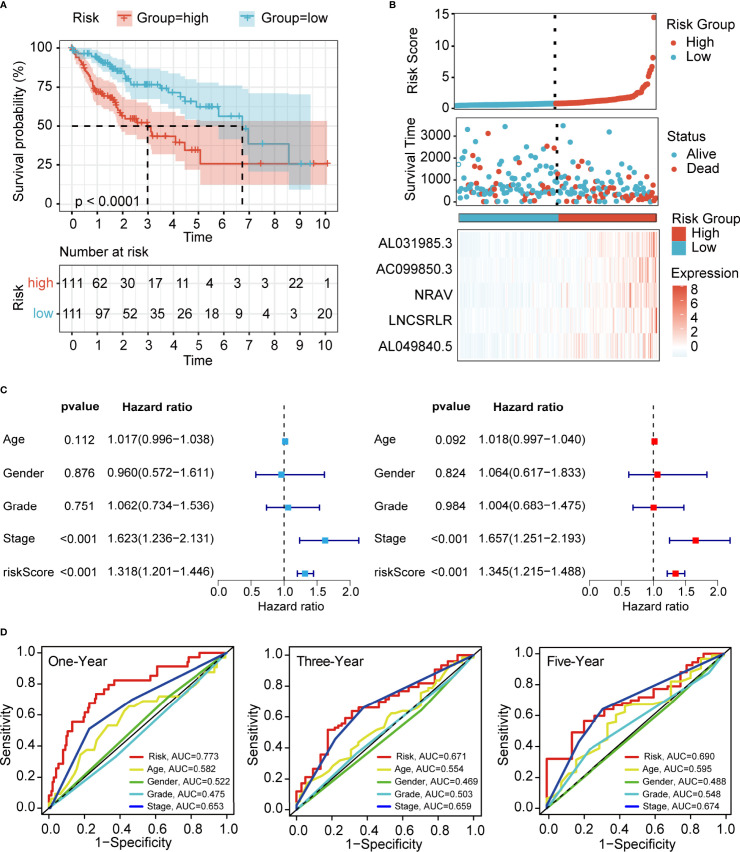
Evaluation of the prognostic value of ferroptosis-related lncRNA signature in the training cohort. **(A)** Kaplan–Meier curves for the OS of patients in different groups (*p* < 0.0001). **(B)** Risk curve of the risk score rank, scatter plot for the survival status distribution, and expression profiles of 5 ferroptosis-associated lncRNAs. **(C)** Univariate and multivariate Cox analyses for the independent prognostic predictor in the training cohort. **(D)** Multivariate ROC curves to predict the sensitivity and specificity of 1-, 3-, and 5-years survival in the training cohort. LncRNA, long non-coding RNA; OS, overall survival; ROC, receiver operating characteristic.

**Figure 4:**
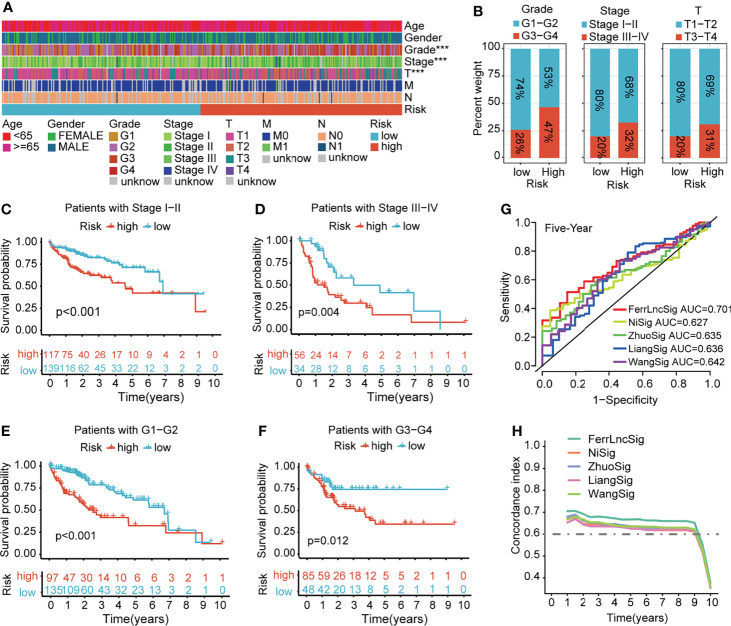
Clinical relevance and model comparison. **(A)** Heatmap of correlations between prognostic signature and clinicopathological features. **(B)** Histograms showing the distribution of grade, stage, and T between the high- and low-risk groups. **(C–F)** Kaplan–Meier analyses of the signature stratified by stage and grade. **(G)** Multivariate ROC curves of different HCC signatures to predict the sensitivity and specificity of 5-year survival. **(H)** Time-dependent c-index curve analysis of different HCC signatures. ROC, receiver operating characteristic; HCC, hepatocellular carcinoma.

To demonstrate the generality of our prognostic model, we applied it to the training cohort and testing cohort. Based on the median risk score of the training cohort, the HCC patients were separated into the high- and low-risk groups. PCA, t-SNE, and UMAP analyses revealed that the signature had the power to clearly separate the different risk groups (**Supplementary Figures 2A, B**). The Kaplan–Meier analysis indicated that patients in the high-risk group had a significantly lower survival probability than their low-risk counterparts (**Figure 3A** and **Supplementary Figure 3A**). Subsequently, we sorted the risk score according to the numerical values, the scatter plot showed that most of the death cases were mainly distributed in the high-risk group, and the heatmap demonstrated that AL049840.5, NRAV, AC099850.3, LNCSRLR, and AL031985.3 were all highly expressed in the high-risk group (**Figure 3B** and **Supplementary Figure 3B**). Univariate Cox regression analysis revealed that the risk score calculated according to 5 ferroptosis-related lncRNAs was related to the OS of HCC patients (**Figure 3C** and **Supplementary Figure 3C** **, left**). After other confounding factors such as age, gender, stage, and grade were adjusted, the multivariate Cox analysis showed likewise that the risk score was related to the prognosis of HCC patients (**Figure 3C** and **Supplementary Figure 3C**, **right**). Furthermore, the time-dependent ROC curves for the risk score and clinicopathological characteristics were generated to compare their predictive power. The area under the curve (AUC) values for 1-, 3-, and 5-year OS of the signature were all above 0.65, which suggested the superior specificity and sensitivity of our signature to other clinical indexes (**Figure 3D** and **Supplementary Figure 3D**). Subsequently, patients in the overall TCGA cohort were classified based on age, gender, grade, TNM stage, and clinical stage for stratification analyses. The clinical heatmap and histogram revealed that patients in the high-risk group significantly had higher tumor grade, larger tumor volume (T stage), and advanced clinical stage (**Figures 4A, B**). The Kaplan–Meier analysis revealed that our signature was still closely associated with worse survival regardless of these clinicopathological features of HCC (**Figures 4C–F** and **Supplementary Figure 4A**). To further assess the predictive efficacy of our signature on OS in HCC patients, we compared it with the other four published TCGA HCC signatures (23, 25–27). The ROC curves and time-dependent c-index proved that our signature was more accurate in prognosis prediction than the four published signatures (**Figures 4G, H** and **Supplementary Figure 4B**). The above results confirmed that our ferroptosis-related lncRNA signature can reliably and accurately serve as an independent prognostic risk factor to predict the survival of HCC patients accurately.

### Construction and Assessment of the Prognostic Prediction Nomogram in Hepatocellular Carcinoma

**Figure 5:**
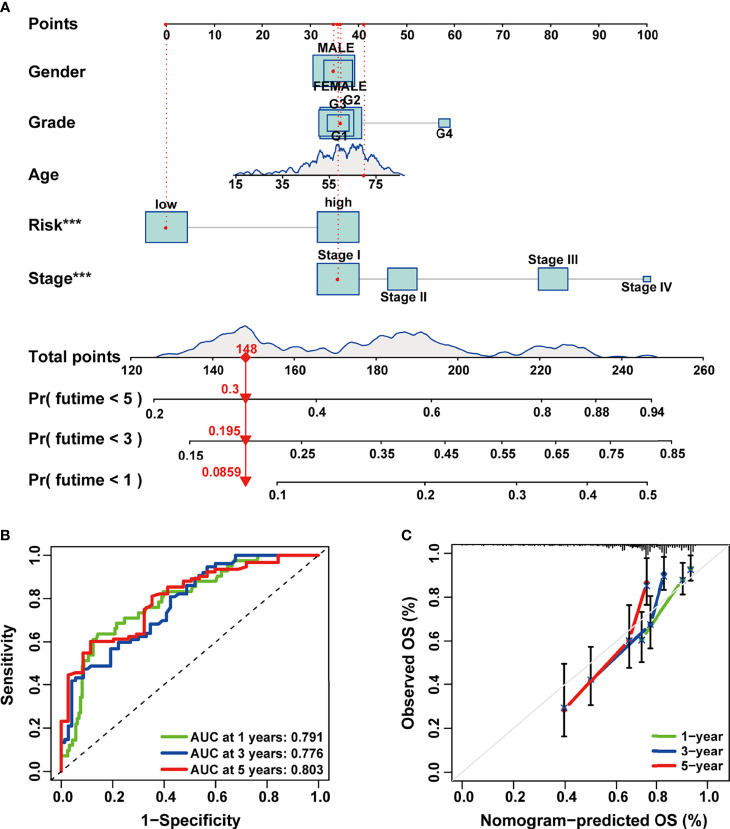
Construction and evaluation of the combined nomogram. **(A)** A prognosis nomogram for predicting 1-, 3-, and 5-year overall survival of HCC patients. **(B)** Time-dependent ROC curves of the nomogram in prediction of prognosis at 1-, 3-, and 5-years point in TCGA HCC cohort. **(C)** The nomogram calibration curves of 1-, 3-, and 5-year survival probabilities. HCC, hepatocellular carcinoma; ROC, receiver operating characteristic; TCGA, The Cancer Genome Atlas.

In order to make our signature applicable in the clinical management of HCC patients, we established a 1-, 3-, and 5-year OS predictive nomogram by integrating the risk score and clinicopathological features of HCC patients (**Figure 5A**). Calibration and ROC curves were used to evaluate the discrimination power and clinical utility of the nomogram. As indicated in the results, the ROC curves demonstrated high predictive accuracy of the nomogram (**Figure 5B**); the predicted lines of the calibration plots for 1-, 3-, and 5-year OS were close to the standard lines (**Figure 5C**). These results suggested that the composite nomogram was capable of predicting the prognosis of HCC patients more precisely than a single clinical feature and our signature, which could assist in clinical decision-making and individualized therapies.

### The Differences in Genomic Landscape and Intratumoral Heterogeneity Between the High- and Low-Risk Groups

**Figure 6:**
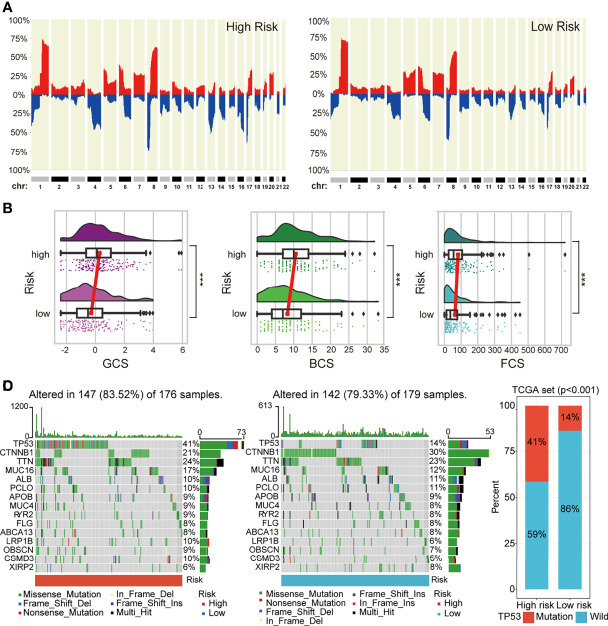
The difference in genomic landscapes between high- and low-risk groups. **(A)** Genome-wide frequency plots showing the overall frequencies of the CNAs in the two risk groups. **(B)** Differences in CNA scores (BCS, FCS, and GCS) between different risk groups. **(C)** Oncoplots of the top 15 significantly mutated genes in different risk groups. **(D)** Histogram showing mutation frequency difference of *TP53* gene between the low- and high-risk groups. CNAs, copy number alterations.

**Figure 7:**
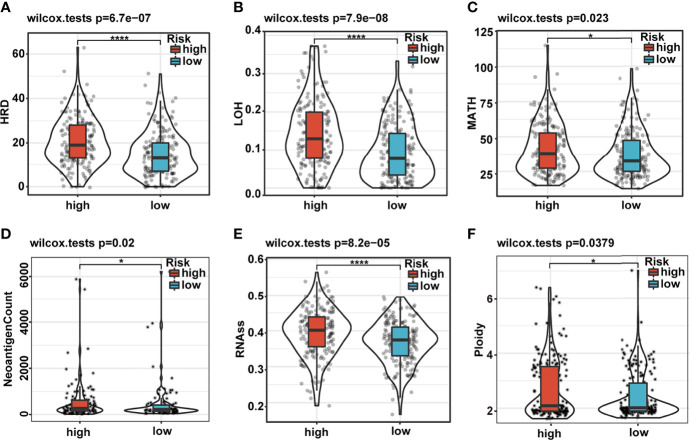
Differences in cancer marker scores between high- and low-risk groups. **(A–F)** The differences in HRD, LOH, MATH, neoantigen count, RNAss, and ploidy, between the low- and high-risk groups, are displayed in boxplots. HRD, homologous recombination deficiency; LOH, loss of heterozygosity; MATH, Mutant-Allele Tumor Heterogeneity.

Genomic CNAs are critical hallmarks of many human cancers with respect to pathogenesis, discrimination, and progression of cancer. Thus, we quantified the CNA burden, including global, broad, and focal CNA scores (GCS, BCS, and FCS, respectively) of each patient using CNApp between the high- and low-risk groups. Interestingly, the genome-wide overall CNA frequencies of patients in the high-risk group were significantly higher than in their low-risk counterparts (red, gain; blue, loss; **Figure 6A**). Similarly, the GCS, BCS, and FCS in the high-risk group were markedly higher than in their low-risk counterparts (**Figure 6B**). Gene mutation was often accompanied by cancer predisposition and tumor progression. The waterfall plot displayed the top 15 significantly mutated genes in the high- and low-risk groups (**Figure 6C**). The mutation frequency of TP53 was significantly higher in the high- than low-risk groups (**Figures 6C, D**). The Kaplan–Meier survival curves indicated that TP53 mutation was a significant unfavorable prognostic factor for survival in TCGA HCC patients (**Supplementary Figure 5A**). We also explored the differences in several critical markers associated with tumorigenesis and cancer progression between the high- and low-risk groups, including LOH, MATH, HRD, tumor neoantigen, ploidy, and RNAss. All these tumor-associated markers in the high-risk group were significantly higher than in their low-risk counterparts and positively correlated with the risk score (**Figures 7A–F** and **Supplementary Figure 5B**). These results suggested that patients in the high-risk group possessed extensive genomic alterations and high tumor heterogeneity, which might be one of the main reasons for their poor prognosis.

### Distinct Tumor Immune Landscape Between the High- and Low-Risk Groups

**Figure 8:**
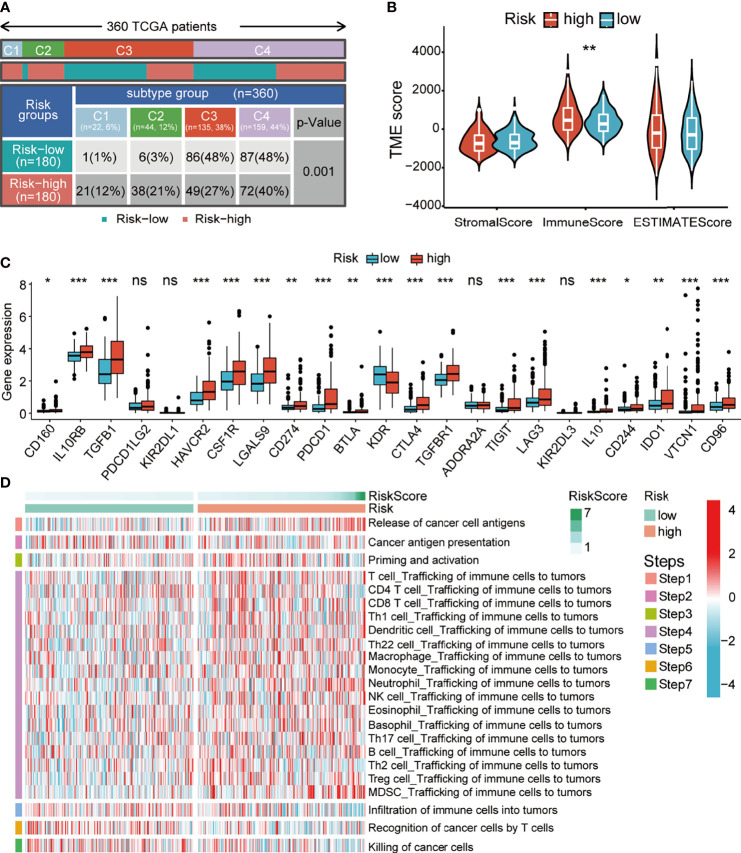
Relationship between the risk score and altered immune landscape. **(A)** Table showing the distribution of HCC immune subtypes between different risk groups. **(B)** Violin plots showing the score differences of tumor microenvironment between the different risk groups. **(C)** Boxplots showing the differential expression of immune-suppressive checkpoints between the different risk groups. **(D)** Heatmap for anticancer immunity cycles pattern. HCC, hepatocellular carcinoma.

Typically, cancer genome alterations have an intricate interplay with the tumor immune microenvironment. Thus, we aimed to investigate the potential relevance between our prognostic signature and the immune status of HCC patients. Firstly, we investigated the correlation between the risk score and the immune subtypes of HCC (C1, wound healing; C2, IFN-γ dominant; C3, inflammatory; and C4, lymphocyte depleted). The C1–4 subtypes were matched since there was no C5 subtype observed, and only one sample was detected in the C6 subtype. Not surprisingly, we found that the C1 and C2 subtypes had a high proportion of high-risk patients (**Figure 8A**). The ESTIMATE analysis of the tumor immune microenvironment showed that the patients of the high-risk group had significantly high immune scores (**Figure 8B**). Thus, we determined whether there was a correlation between the expression of inhibitory immune checkpoints and the risk score. Surprisingly, the expression levels of almost all checkpoints were significantly upregulated in the high-risk group (**Figure 8C**). Finally, we explored the differences in the anticancer immunity cycle patterns between the two risk groups. Seven‐step immune activity scores in the two risk groups are presented in the line chart and heatmap. The results showed that the scores of step 1 (releasing cancer cell antigen) and step 4 (trafficking of immune cells to tumors) were higher in the high-risk group, while the scores of step 5 (infiltration of immune cells into tumors), step 6 (recognition of cancer cells by T cells), and step 7 (killing cancer cells) were higher in the low-risk group (**Figure 8D** and **Supplementary Figure 6A**). In addition, correlation analysis showed that the risk score had a significant positive correlation with the recruitment of Th17 cells, Th22 cells, regulator T cells (Tregs), and myeloid-derived suppressor cells (MDSCs) (**Supplementary Figure 6B**). Importantly, the scores of steps 5, 6, and 7 could significantly impact the OS of HCC patients, which might explain the reason for survival outcomes between the two risk groups. The above results suggested that our signature might have a significant impact on the immune status of HCC patients.

### Differences in Potential Biological Pathways Related to the Risk Score

**Figure 9:**
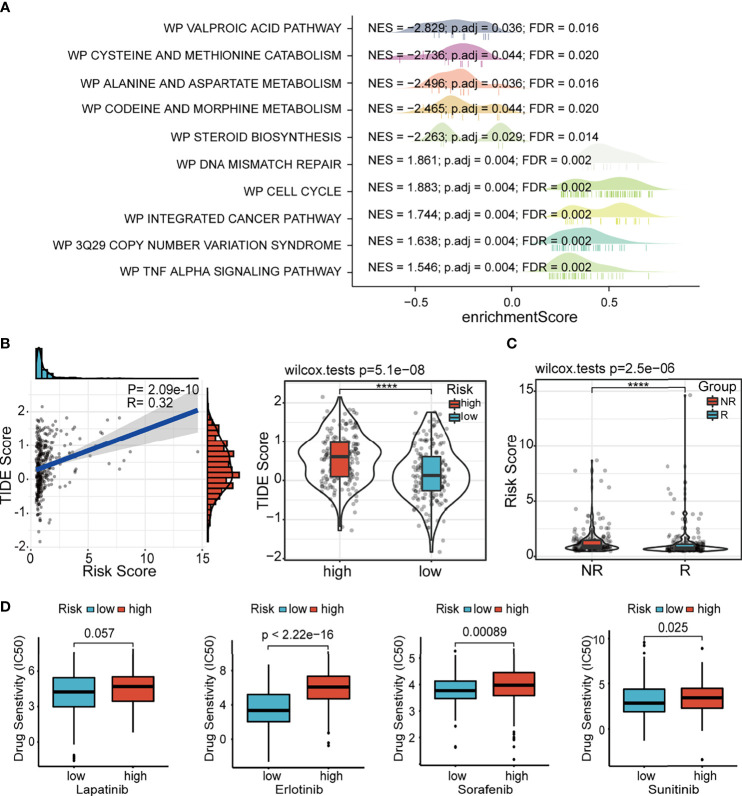
GSEA and drug susceptibility analysis. **(A)** GSEA of ferroptosis-associated lncRNA signature based on WikiPathways database. **(B)** Correlation between the risk score and the TIDE score. **(C)** Correlation between the risk score and immune checkpoint blockade response. GSEA, gene set enrichment analysis; lncRNA, long non-coding RNA.

In order to clarify the potential biological pathways related to our signature, we conducted GSEA based on the MSigDB gene sets, including WikiPathways and Kyoto Encyclopedia of Genes and Genomes (KEGG) pathway (**Figure 9A** and **Supplementary Figure 7**); these enrichment results were consistent. The immune-, genomic alteration-, cell cycle-, and other classical cancer-related pathways, such as primary immunodeficiency, DNA mismatch repair, 3q29 CNV syndrome, cell cycle, and p53 signaling pathway, were mainly activated in the high-risk group. Conversely, the patients in the low-risk group revealed significant activation of many metabolic pathways. These functional enrichment results also corroborated the correlations of our signature with immune microenvironment and genomic heterogeneity analyzed in previous sections.

### Distinct Drug Sensitivity Between the High- and Low-Risk Groups

Considering the potential differences in immune status and genomic landscape between the two risk groups, we further explored the difference in the immunotherapy response between the high- and low-risk groups. The results showed that the TIDE score was significantly higher in the high-risk group and positively associated with the risk score, which revealed that patients in the low-risk group might be more responsive to immunotherapy than their high-risk counterparts (**Figure 9B**). The results predicted by ImmuCellAI showed that the risk score of patients in the non-response (NR) group was higher than patients in the response (R) group (**Figure 9C**). Additionally, the susceptibility to various chemotherapeutic drugs commonly used in HCC varied between the two risk groups. Similar to the results of immunotherapy response, we found that all these drugs, including lapatinib, erlotinib, sorafenib, and sunitinib, had low IC50 values in the low-risk group, indicating the sensitivity of the patients to chemotherapy (**Figure 9D**). Thus, it can be speculated that our signature guides the clinical decisions for HCC patients.

### Effect of LNCSRLR on Malignant Biological Behavior of Hepatocellular Carcinoma Cells

**Figure 10:**
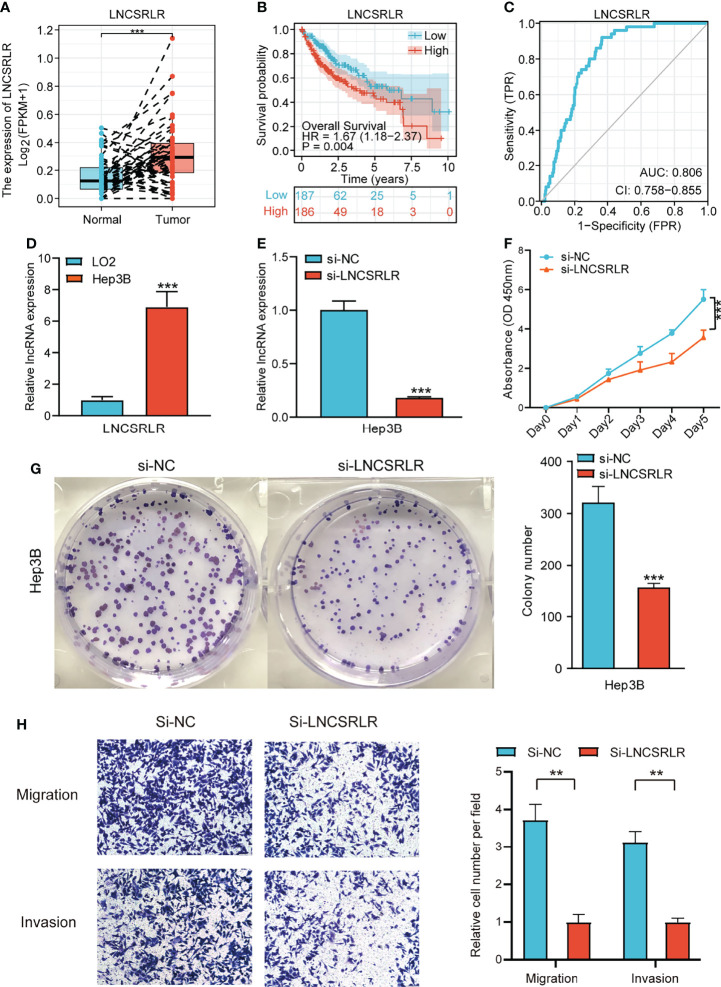
The role of LNCSRLR in HCC. **(A)** Differential expression analysis of LNCSRLR in paired samples of TCGA HCC cohort. **(B)** Kaplan–Meier analytical evaluation of the prognostic values of LNCSRLR. **(C)** ROC curve of LNCSRLR for assessing the diagnostic performance in HCC. **(D)** RT-qPCR result of LNCSRLR in normal human hepatic cell line (LO2) and HCC cell line (Hep3B). **(E)** RT-qPCR validation of downregulation by siRNA. **(F)** CCK-8 cell proliferation curve. **(G)** Self-renewal ability was detected by plate-cloning experiments. **(H)** Transwell migration and invasion assays. HCC, hepatocellular carcinoma; TCGA, The Cancer Genome Atlas; ROC, receiver operating characteristic.

LNCSRLR has the highest coefficient in the model, and hence, we studied its role in HCC. Paired sample t-test of TCGA cohort showed that LNCSRLR expression was upregulated in HCC (**Figure 10A**). The Kaplan–Meier survival curve confirmed that the high expression of LNCSRLR showed an adverse prognosis (**Figure 10B**). In addition, the ROC curve indicated a high diagnostic value of LNCSRLR in HCC (**Figure 10C**). Then, we used a series of functional experiments *in vitro* to verify the role of LNCSRLR in the HCC cell line. qRT-PCR results showed that expression levels of LNCSRLR in Hep3B cells were higher than in LO2 cells (**Figure 10D**). RNAi and control were transfected into Hep3B cells. The relative lncRNA levels were measured by qRT-PCR (**Figure 10E**). The CCK-8 assay evaluated the proliferation effect of LNCSRLR, and the data showed that si-LNCSRLR suppressed the proliferation rate of cells (**Figure 10F**). Downregulation of LNCSRLR decreased the clone formation ability and inhibited cell growth (**Figure 10G**). The scratch assay showed that si-LNCSRLR decreased the Hep3B cell migration capacity compared to the control group (**Supplementary Figure 8**). The data on migration and invasion showed that si-LNCSRLR lowered the cell migration and invasion ability (**Figure 10H**). These results suggested a key role of LNCSRLR in the occurrence and progression of HCC.

## Discussion

In the present study, we explored the differences in ferroptosis activity between tumor and normal samples using multiple datasets in HCC. A novel prognostic model, including five ferroptosis-related lncRNAs, was established and validated in TCGA HCC dataset, which demonstrated a good degree of discrimination and calibration in predicting survival. Furthermore, various bioinformatics analyses confirmed striking differences in the genomic landscape, immune status, and drug sensitivity between the high- and low-risk groups. These findings revealed the dysregulation and critical role of ferroptosis-related lncRNAs in HCC.

Although ferroptosis is likely to occur in tumor cells, it often exhibits enhanced resistance to ferroptosis *via* altered gene expression (28). In this study, we confirmed the pathway-level differences in ferroptosis between the normal and tumor samples in HCC patients using multiple datasets and gene sets. Surprisingly, the scores of ferroptosis drivers and ferroptosis suppressors showed an opposite trend. These results were similar to the above studies indicating that the ferroptosis process is inhibited in HCC tissue samples. Previous studies have shown that the prognostic lncRNA signatures can predict survival in a variety of human cancers, such as breast cancer (29) and gastric cancer (30). The correlation and differential expression analyses in TCGA HCC dataset revealed about 61% (781/1271) of ferroptosis-related lncRNAs as the differentially expressed ferroptosis-related lncRNAs. Subsequently, five lncRNAs (AL049840.5, NRAV, AC099850.3, LNCSRLR, and AL031985.3) were included in our prognostic model after LASSO regression and Cox regression analysis. Some of these five candidate lncRNAs are closely related to cancer development and prognosis in several studies. Mou et al. (31) revealed that lncRNA AC099850.3 was significantly highly expressed in NSCLC tissues and cells, closely related to the development and procession of NLSCL. Sun et al. (29) showed that lncRNAs AL031985.3 and NRAV promote cancer progression and are highly expressed in four HCC cell lines. Another study reported that the upregulated expression of LNCSRLR in renal cell carcinoma patients was significantly correlated with sorafenib resistance (32). However, the role of lncRNA AL049840.5 in tumors has not yet been reported and needs to be investigated further. This evidence supports our bioinformatics results that the five hub lncRNAs play critical roles in cancer pathology and malignant progression.

Genomic alterations and intratumor heterogeneity are the hallmarks of cancer. Based on our signature and previous studies, the patients in the high-risk group had extensive genomic alterations and high tumor heterogeneity, reflected in the indicators including high CNA scores, high TP53 mutation frequencies, high LOH scores, high HRD scores, high MATH scores, high RNAss, high ploidy, and high tumor neoantigen count. Survival analysis indicated that high TP53 mutation frequency is one of the major causes of poor prognosis in the high-risk group. These indicators are closely related to the unfavorable prognosis of multiple cancers (33–37). Genome alterations have intricate connections with the tumor immune microenvironment. Although the potential mechanisms of ferroptosis susceptibility have recently been investigated in-depth, the correlation between ferroptosis and anticancer immunity remains elusive. In immunity module analysis, we observed that patients of different risk stratification have a significantly distinct immune microenvironment and distribution of immune subtypes. These results indicated a significant difference in immune status between patients of different groups. One unanticipated result was that the expression of almost all inhibitory immune checkpoints was significantly upregulated in the high-risk group. In the analysis of anticancer immunity cycles, we found that patients in the high-risk group recruited several immunosuppressive cells, including Th17 cells, Th22 cells, Tregs, and MDSCs. Thus, immune cells of patients in the low-risk group easily infiltrate the tumors and the cancer cells that could be identified and killed by T cells effectively. Pathway enrichment analyses based on different databases were consistent and confirmed the results of genome and immunity modules. Therefore, it could be postulated that the immunosuppressive tumor microenvironment caused by genomic disorders is one of the major reasons for the extremely unfavorable prognosis of patients in the high-risk group. Finally, considering that the immune status of patients is associated with drug reactions, and the selective induction of ferroptosis has been adopted as a potential treatment strategy in some kinds of cancer, we predicted drug sensitivity with respect to immune checkpoint inhibitors and commonly used chemotherapeutics in HCC. The results showed that patients in the low-risk group were susceptible to both immunotherapy and chemotherapy. Previous studies have shown that capecitabine can induce ferroptosis among patients with metastatic breast cancer (38). Not only that, multiple studies have shown that HCC patients undergoing sorafenib failure were found to be safe and effective when taking capecitabine (39–41), implying that capecitabine might be a potential second-line treatment for HCC after sorafenib discontinuation. Therefore, it could be deduced that capecitabine may be a more effective treatment option for HCC patients in the high-risk groups.

LNCSRLR is the molecule with the highest regression coefficient in our signature; thus, we conducted an in-depth study on its role in HCC. The paired sample t-test and RT-qPCR confirmed that the expression of LNCSRLR was upregulated in HCC samples and HCC cell lines. ROC curve analysis and the Kaplan–Meier survival curves confirmed the value of LNCSRLR in the diagnosis and survival prediction of HCC patients. In addition, efficient LNCSRLR silencing strongly inhibited *in vitro* proliferation, migration, and invasion of Hep3B cells. These results indicated that LNCSRLR might be involved in the occurrence and development of liver cancer as a tumor-promoting factor.

Nevertheless, the present study has several limitations. Firstly, our prognostic model could not be validated in external HCC cohorts because the ICGC-LICA and GSE40144 cohorts lack sufficient survival time data or survival status information, while other HCC datasets, such as ICGC-LIRI and GSE14520, did not contain any or only a few selected genes. Secondly, this was a retrospective study, and thus, additional prospective real-world analysis is required to verify the clinical application of this approach.

## Conclusion

In summary, a novel prognostic model based on 5 ferroptosis-related lncRNAs in HCC was constructed and validated. This signature could be utilized as an efficient computational technique for predicting the clinical prognosis of patients with HCC, which was robustly connected to the genomic heterogeneity and immunosuppressive status of patients, thereby providing an option for drug therapy. Hence, our findings provided some new perspectives for prognostic evaluation and were expected to be a potential biomarker in HCC.

## Data Availability Statement

Publicly available datasets were analyzed in this study. These data can be found in the article/**Supplementary Material**.

## Author Contributions

GW, QL, and JF conceptualized and designed this study. GL, YL, and YZ conducted the experiments. GL, YZ, JZ, and YX collected and analyzed the relevant data. GL, YL, and XW wrote the manuscript. JZ, ZZ, XW, YX, CZ, and YL designed and finalized the tables and figures. YZ, CZ, and JF reviewed and corrected the manuscript. All authors read and approved the final manuscript.

## Funding

This work was supported by the National Natural Science Foundation of China [grant nos. 81872184 and 81773031].

## Conflict of Interest

The authors declare that the research was conducted in the absence of any commercial or financial relationships that could be construed as a potential conflict of interest.

## Publisher’s Note

All claims expressed in this article are solely those of the authors and do not necessarily represent those of their affiliated organizations, or those of the publisher, the editors and the reviewers. Any product that may be evaluated in this article, or claim that may be made by its manufacturer, is not guaranteed or endorsed by the publisher.
