# Supplementary material for: A Novel Ferroptosis-Related Long Non-Coding RNA Prognostic Signature Correlates With Genomic Heterogeneity, Immunosuppressive Phenotype, and Drug Sensitivity in Hepatocellular Carcinoma
Source: Front Immunol. 2022 Jul 8;13:929089. doi: 10.3389/fimmu.2022.929089 (PMC9304774; doi:10.3389/fimmu.2022.929089)
Supplement: Supplementary file 1 [file DataSheet_1.pdf]

Supplementary Material

1 SUPPLEMENTARY DATA

Download link sharing scope set to "anyone" is not available

Please obtain the original data and code through the following account number

Account：drlgh1124@sina.com Password: z5227218

https://www.jianguoyun.com/p/DdYUFfUQ_e7BChiro7oEIAA

2 SUPPLEMENTARY FIGURES AND TABLES

2.1 SUPPLEMENTARY FIGURES

Figure S1

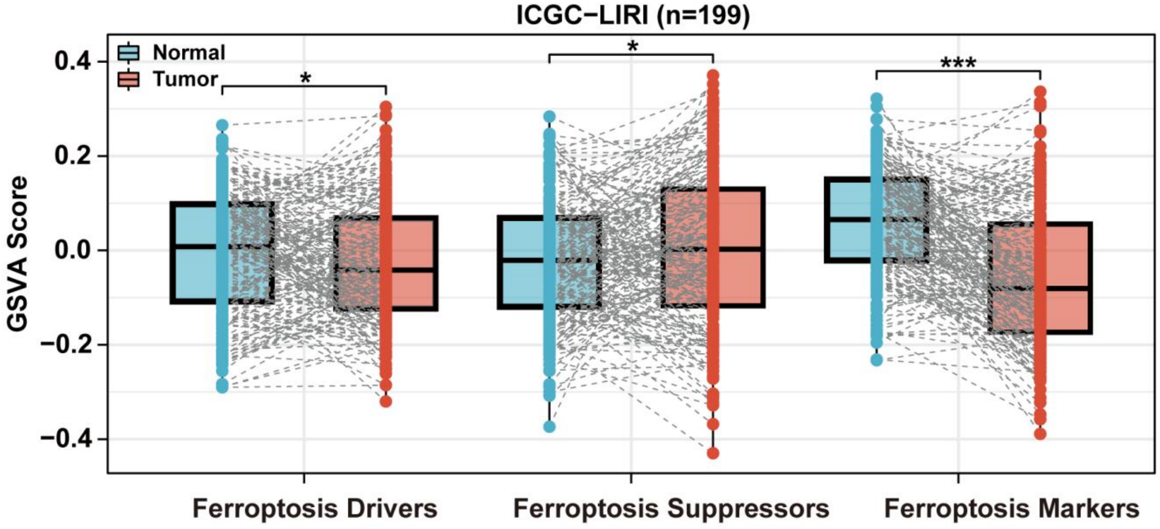

Supplementary Figure 1. GSVA scores of ferroptosis drive, ferroptosis suppressor and ferroptosis maker in paired samples of ICGC-LIRI cohort.

Figure S2

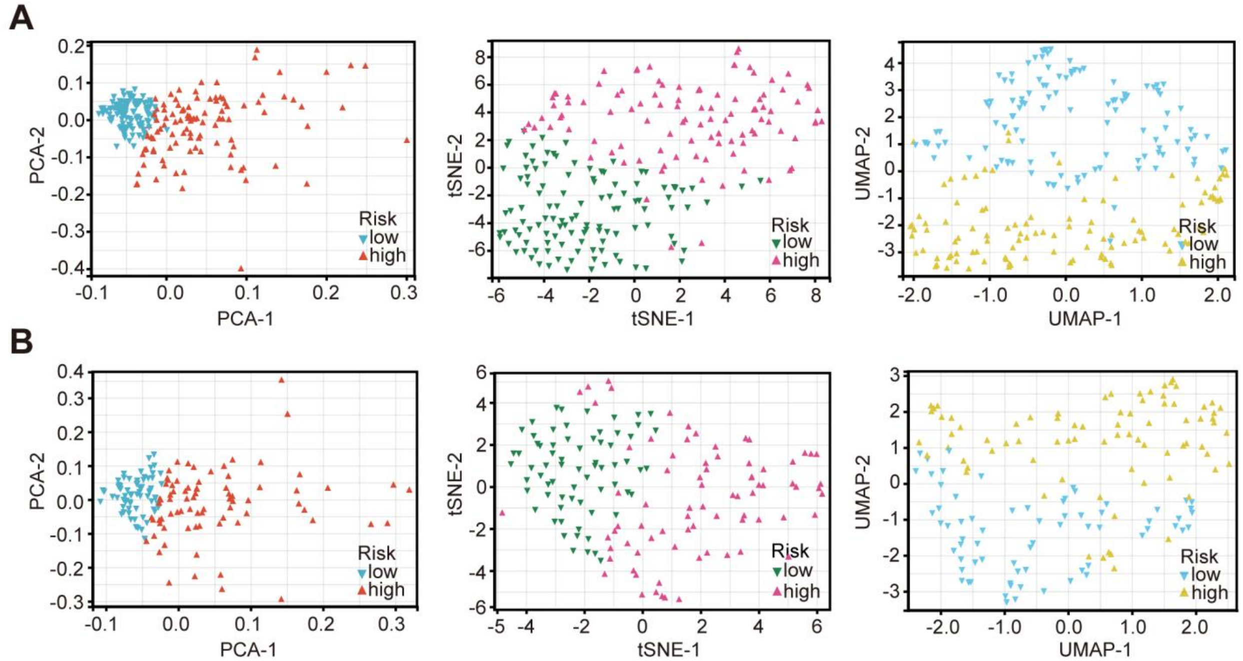

Supplementary Figure 2. (A) PCA, t-SNE and UMAP analysis of patients in training cohort based on the expression profiles of the 5 signature lncRNAs. **(B)** PCA, t-SNE and UMAP analysis of patients in testing cohort based on the expression profiles of the 5 signature lncRNAs.

Figure S3

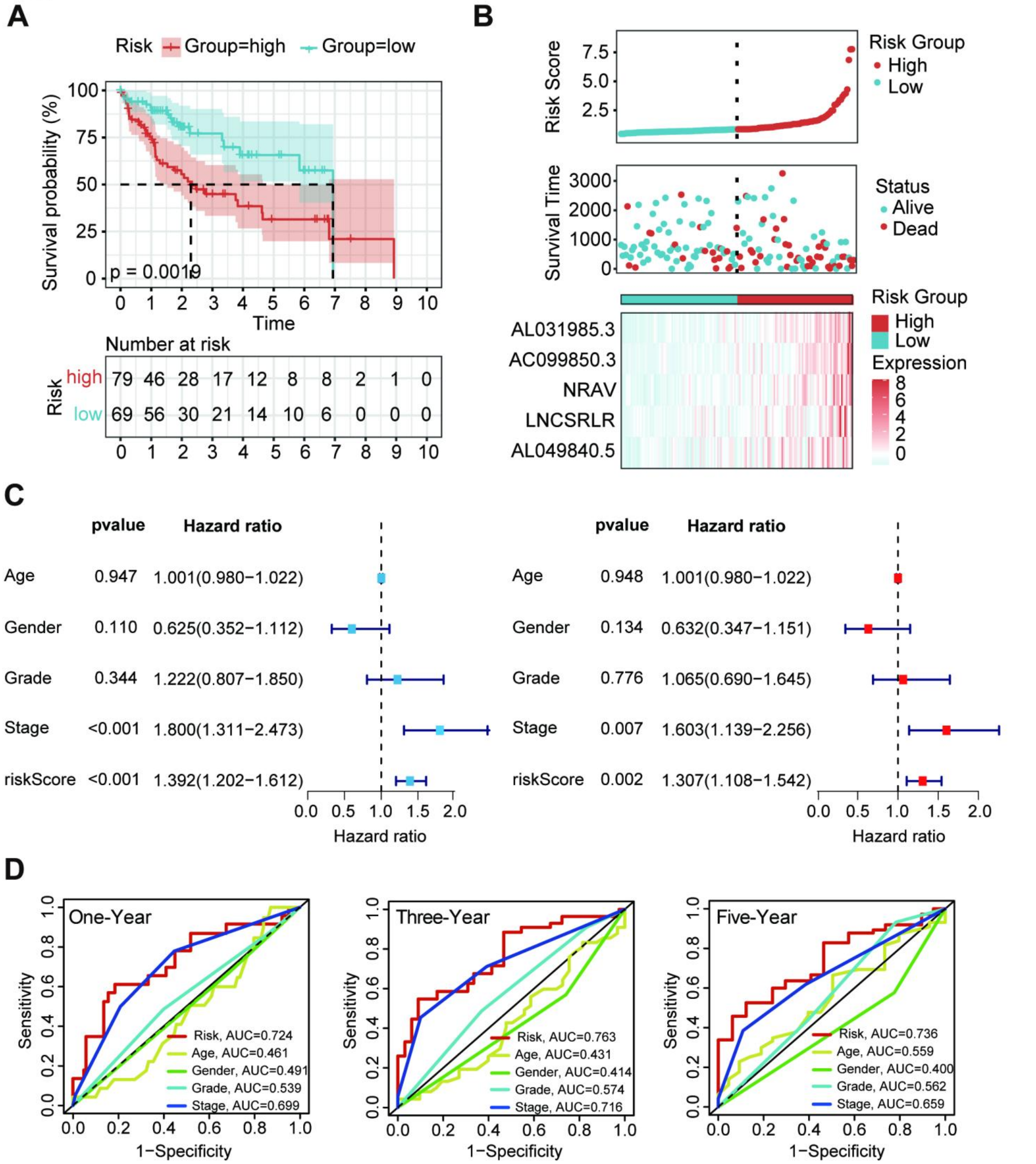

Supplementary Figure 3. (A) Kaplan-Meier curves for the OS of patients in different groups

(p=0.0019). **(B)** Risk curve of the risk score rank, scatter plot for the survival status distribution, and expression profiles of 5 ferroptosis-associated lncRNAs. **(C)** Univariate and multivariate Cox analyses for the independent prognostic predictor in the testing cohort. **(E)** Multivariate ROC curves to predict the sensitivity and specificity of 1, 3, and 5 years survival in the testing cohort.

Figure S4

A

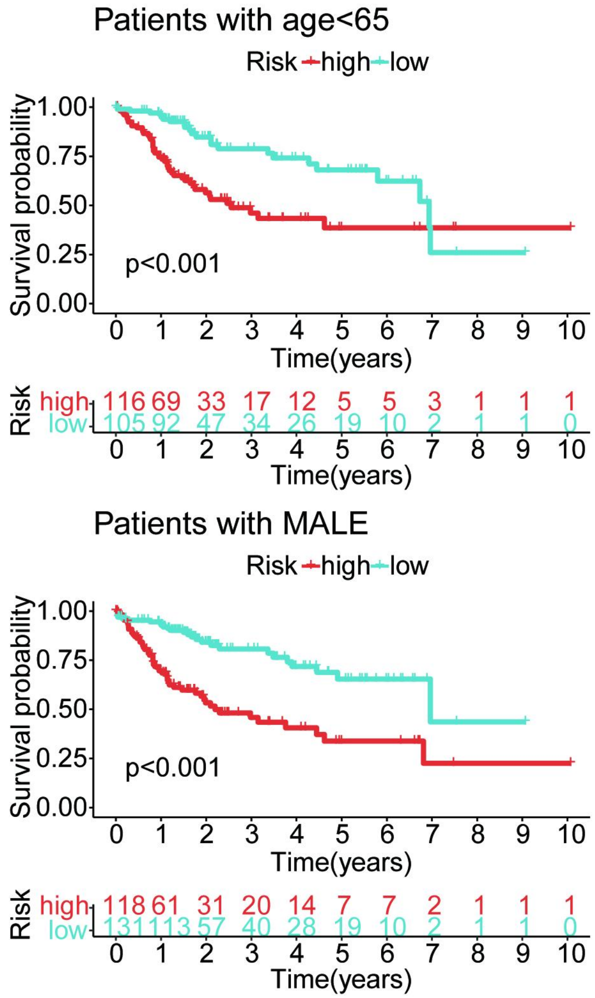

Patients with age<65
Risk high low
Survival probability
1.00
0.75
0.50
0.25
0.00
p<0.001
Time(years)
Risk
high 116 69 33 17 12 5 5 3 1 1 1
low 105 92 47 34 26 19 10 2 1 1 0
Patients with age>=65
p=0.003
high 69 39 25 17 11 7 6 2 2 1 0
low 80 61 35 22 14 9 5 2 2 1 0
Patients with MALE
p<0.001
high 118 61 31 20 14 7 7 2 1 1 1
low 131 113 57 40 28 19 10 2 1 1 0
Patients with FEMALE
p=0.004
high 67 47 27 14 9 5 4 3 2 1 0
low 54 40 25 16 12 9 5 2 2 1 0

B

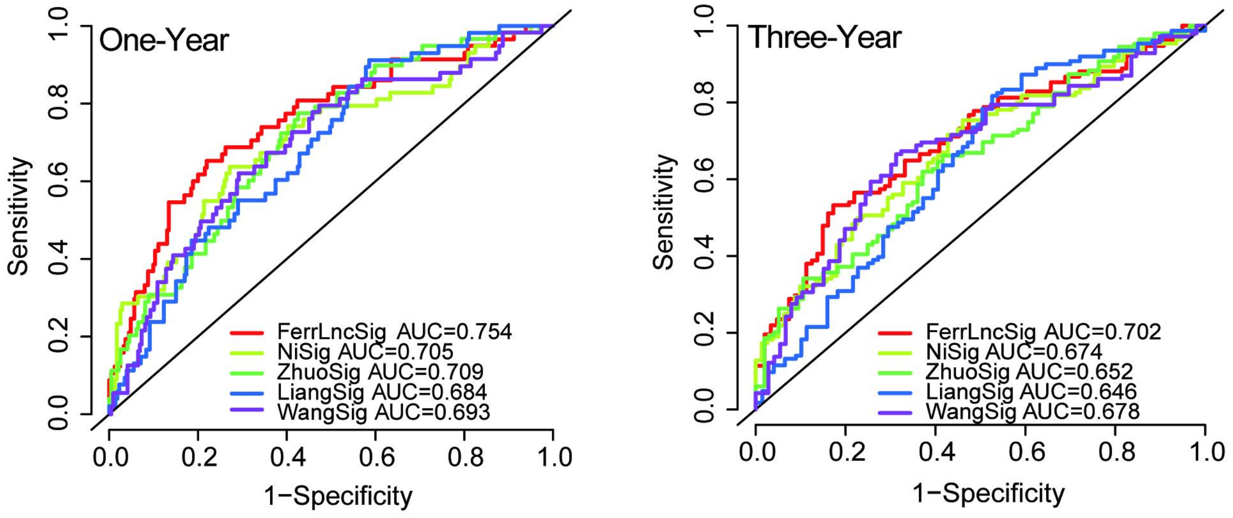

One-Year
Sensitivity
1−Specificity
FerrLncSig AUC=0.754
NiSig AUC=0.705
ZhuoSig AUC=0.709
LiangSig AUC=0.684
WangSig AUC=0.693
Three-Year
FerrLncSig AUC=0.702
NiSig AUC=0.674
ZhuoSig AUC=0.652
LiangSig AUC=0.646
WangSig AUC=0.678

Supplementary Figure 4. (A) Kaplan-Meier analyses of the signature stratified by age and gender. **(B)** Multivariate ROC curves of different HCC signatures to predict the sensitivity and specificity of 1 and 3 years survival.

Figure S5

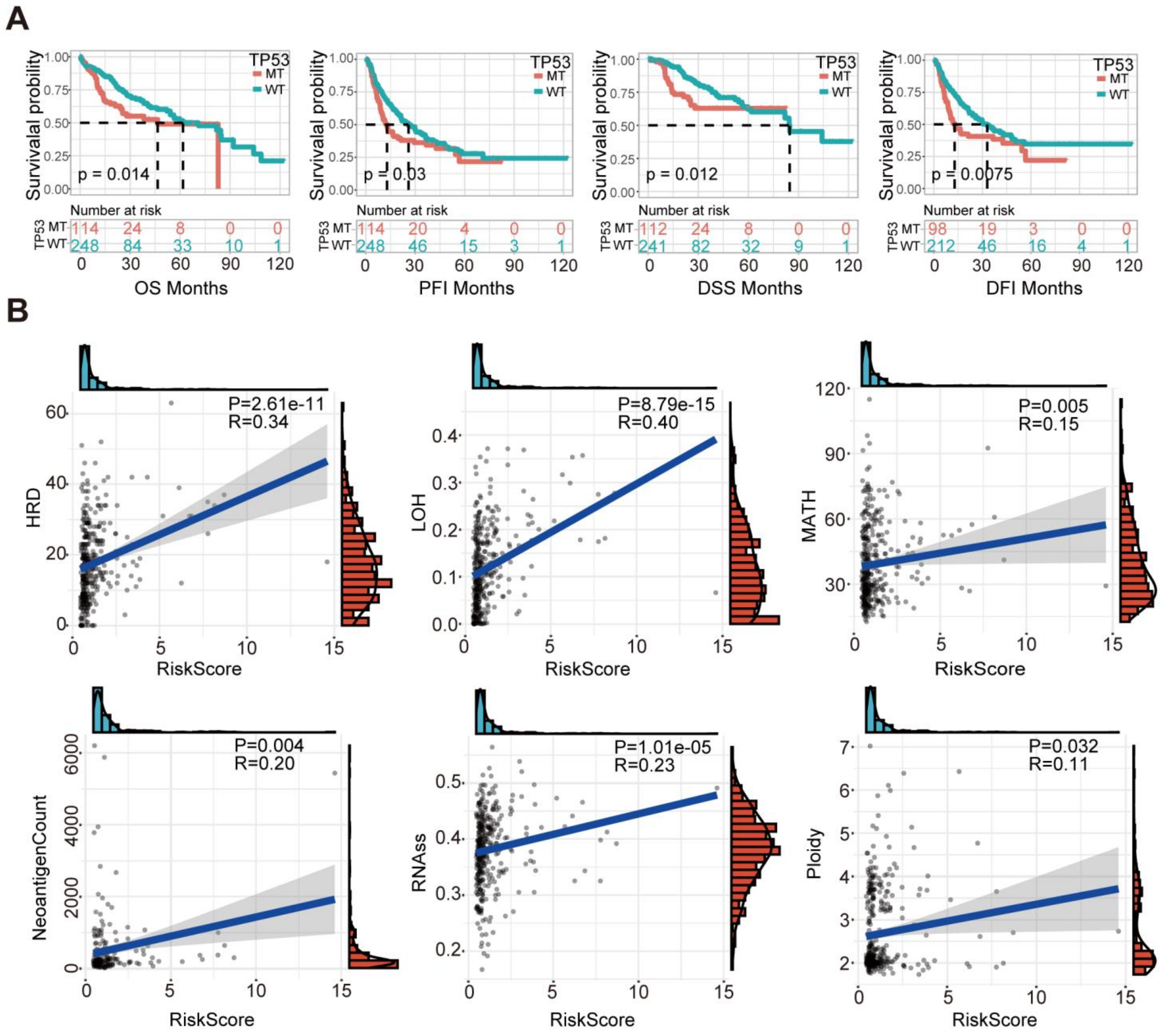

Supplementary Figure 5. (A) OS, PFI, DSS, and DFI of patients stratified by TP53 mutation status in TCGA-LIHC cohort. WT, wild-type TP53; Mut, TP53 mutation. OS, overall survival; DSS, disease-specific survival; PFI, progression-free interval; DFI, disease-free interval. **(B)** Correlations between risk score and HRD, LOH, MATH, ploidy, neoantigen count and RNAss.

Figure S6

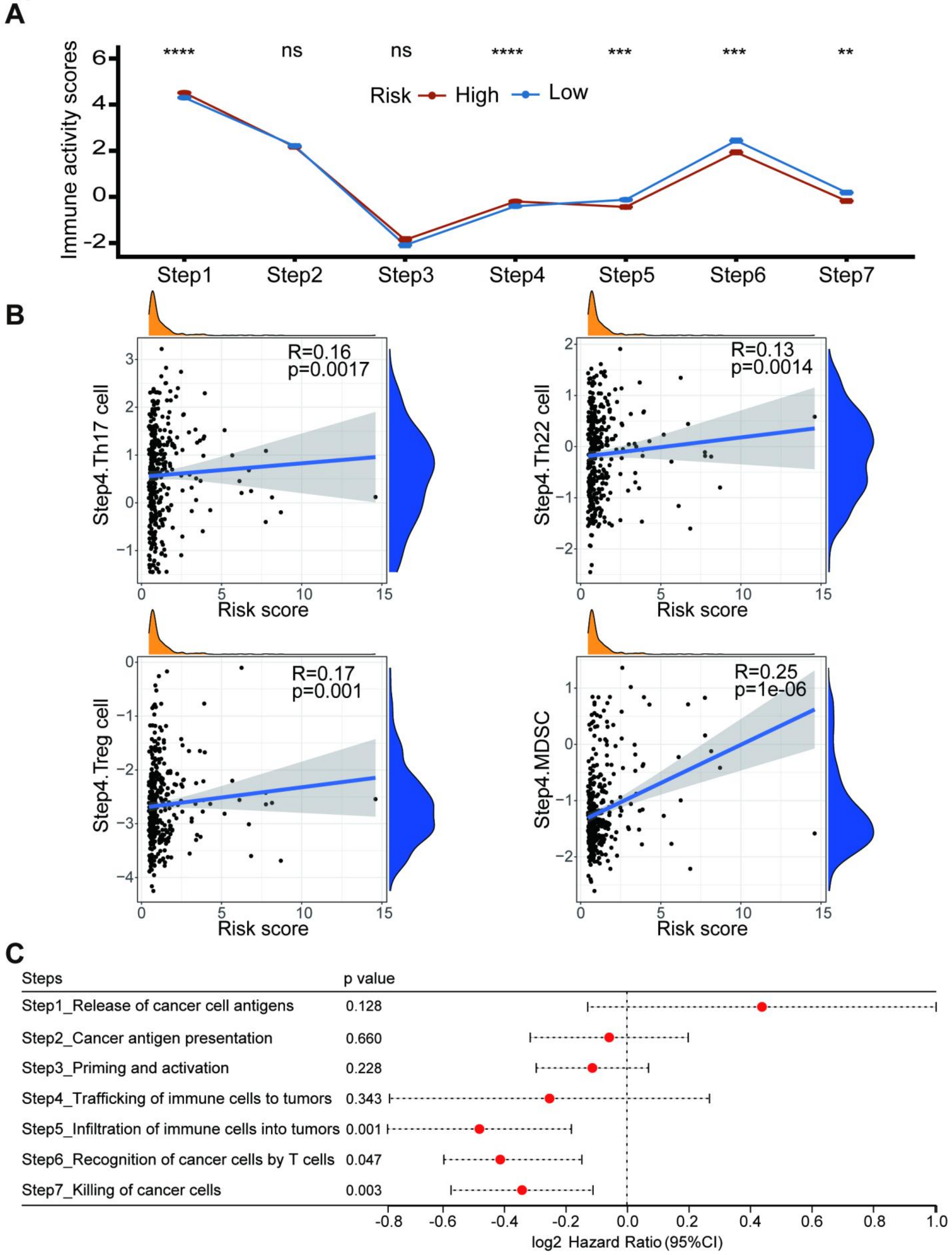
A
Immune activity scores
**** ns ns **** *** *** **
Risk High Low
Step1 Step2 Step3 Step4 Step5 Step6 Step7
B
Step4.Th17 cell
R=0.16
p=0.0017
Risk score
Step4.Th22 cell
R=0.13
p=0.0014
Risk score
Step4.Treg cell
R=0.17
p=0.001
Risk score
Step4.MDSC
R=0.25
p=1e-06
Risk score
C
Steps
p value
Step1_Release of cancer cell antigens 0.128
Step2_Cancer antigen presentation 0.660
Step3_Priming and activation 0.228
Step4_Trafficking of immune cells to tumors 0.343
Step5_Infiltration of immune cells into tumors 0.001
Step6_Recognition of cancer cells by T cells 0.047
Step7_Killing of cancer cells 0.003
log2 Hazard Ratio (95%CI)

Supplementary Figure 6. (A) Line chart showing the differences of seven-step cancer-immunity cycle between the different risk groups. **(B)** Correlations between risk score and the cell recruitments of Th17, Th22, Treg and MDSC. **(C)** Survival analysis of seven-step cancer-immunity cycle.

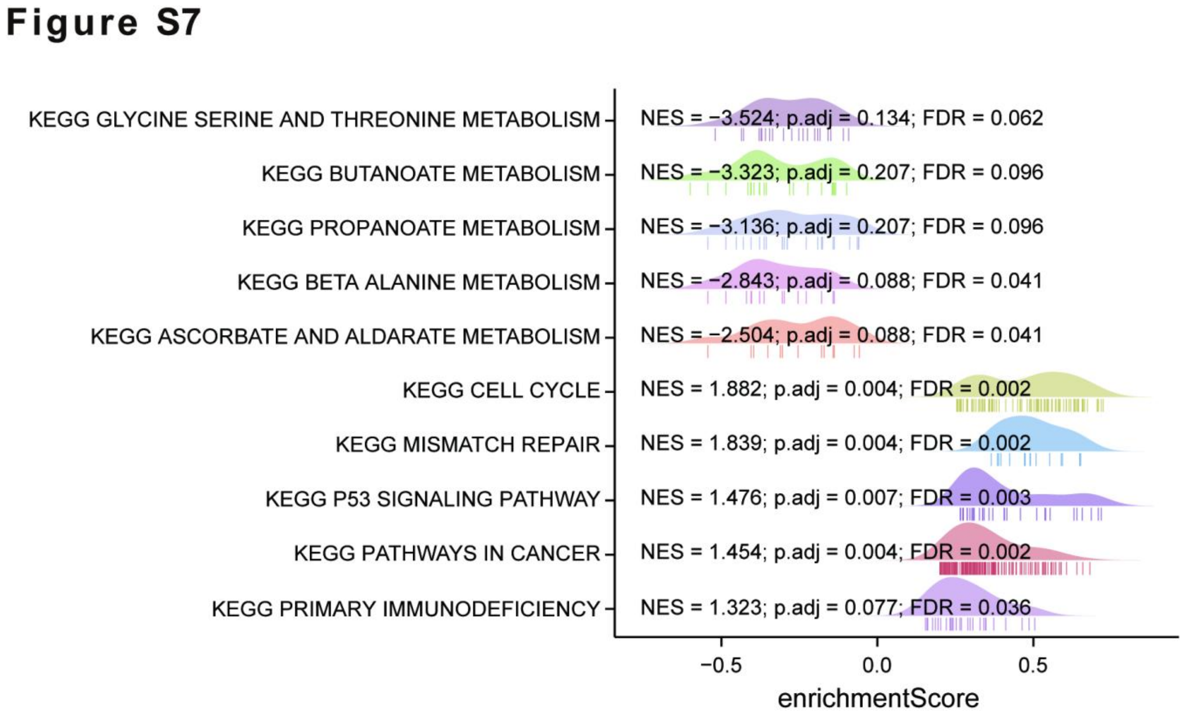

Supplementary Figure 7. Gene set enrichment analysis of ferroptosis-associated lncRNA signature based on KEGG database.

Figure S8

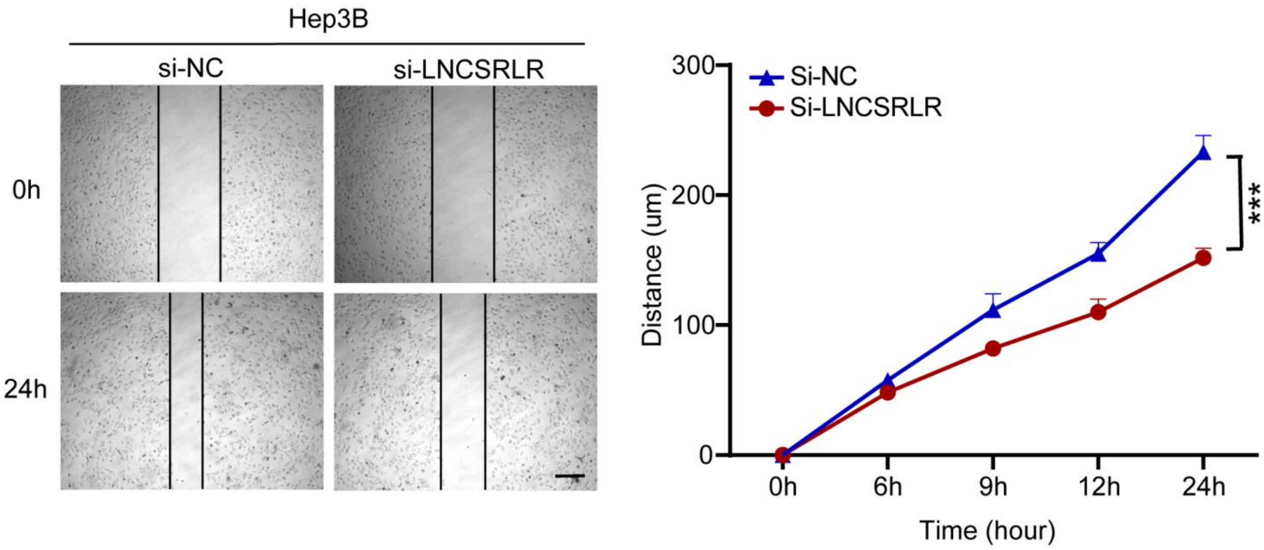

Supplementary Figure 8. Migration was determined by scratch experiments.

2.2 SUPPLEMENTARY TABLES

Supplementary Figure 1. ferroptosis-associated genes obtained from the FerrDb website.

Symbol	Name
PTGS2	Prostaglandin-endoperoxide synthase 2
DUSP1	Dual specificity phosphatase 1
NOS2	Nitric oxide synthase 2
NCF2	Neutrophil cytosolic factor 2
MT3	Metallothionein 3
UBC	Ubiquitin C
ALB	Albumin
TXNRD1	Thioredoxin reductase 1

SRXN1	Sulfiredoxin 1
GPX2	Glutathione peroxidase 2
BNIP3	BCL2 interacting protein 3
OXSR1	Oxidative stress responsive kinase 1
SELENOS	Selenoprotein S
ANGPTL7	Angiopoietin like 7
CHAC1	ChaC glutathione specific gamma-glutamylcyclotransferase 1
SLC7A11	Solute carrier family 7 member 11
DDIT4	DNA damage inducible transcript 4
LOC284561	_NA_
ASNS	Asparagine synthetase (glutamine-hydrolyzing)
TSC22D3	TSC22 domain family member 3
DDIT3	DNA damage inducible transcript 3
JDP2	Jun dimerization protein 2
SESN2	Sestrin 2
SLC1A4	Solute carrier family 1 member 4
PCK2	Phosphoenolpyruvate carboxykinase 2, mitochondrial
TXNIP	Thioredoxin interacting protein
VLDLR	Very low density lipoprotein receptor
GPT2	Glutamic--pyruvic transaminase 2

PSAT1	Phosphoserine aminotransferase 1
LURAP1L	Leucine rich adaptor protein 1 like
SLC7A5	Solute carrier family 7 member 5
HERPUD1	Homocysteine inducible ER protein with ubiquitin like domain 1
XBP1	X-box binding protein 1
ATF3	Activating transcription factor 3
SLC3A2	Solute carrier family 3 member 2
CBS	Cystathionine beta-synthase
ATF4	Activating transcription factor 4
ZNF419	Zinc finger protein 419
KLHL24	Kelch like family member 24
TRIB3	Tribbles pseudokinase 3
ZFP69B	ZFP69 zinc finger protein B
ATP6V1G2	ATPase H+ transporting V1 subunit G2
VEGFA	Vascular endothelial growth factor A
GDF15	Growth differentiation factor 15
TUBE1	Tubulin epsilon 1
ARRDC3	Arrestin domain containing 3
CEBPG	CCAAT enhancer binding protein gamma
SNORA16A	Small nucleolar RNA, H/ACA box 16A

RGS4	Regulator of G protein signaling 4
BLOC1S5-TXNDC5	BLOC1S5-TXNDC5 readthrough (NMD candidate)
LOC390705	_NA_
EIF2S1	Eukaryotic translation initiation factor 2 subunit 1
KIM-1	Kidney injury molecule-1
IL6	Interleukin 6
CXCL2	C-X-C motif chemokine ligand 2
RELA	RELA proto-oncogene, NF-kB subunit
HSD17B11	Hydroxysteroid 17-beta dehydrogenase 11
AGPAT3	1-acylglycerol-3-phosphate O-acyltransferase 3
SETD1B	SET domain containing 1B, histone lysine methyltransferase
HMOX1	Heme oxygenase 1
TF	Transferrin
FTL	Ferritin light chain
RPL8	Ribosomal protein L8
ATP5MC3	ATP synthase membrane subunit c locus 3
TFRC	Transferrin receptor
MAFG	MAF bZIP transcription factor G
IL33	Interleukin 33
FTH1	Ferritin heavy chain 1

SLC40A1	Solute carrier family 40 member 1
TF	Transferrin
TFRC	Transferrin receptor
FTH1	Ferritin heavy chain 1
GPX4	Glutathione peroxidase 4
HAMP	Hepcidin antimicrobial peptide
HSPB1	Heat shock protein family B (small) member 1
NFE2L2	Nuclear factor, erythroid 2 like 2
STEAP3	STEAP3 metalloreductase
DRD5	Dopamine receptor D5
GPX4	Glutathione peroxidase 4
DRD4	Dopamine receptor D4
MAP3K5	Mitogen-activated protein kinase kinase kinase 5
MAPK14	Mitogen-activated protein kinase 14
SLC2A1	Solute carrier family 2 member 1
SLC2A3	Solute carrier family 2 member 3
SLC2A6	Solute carrier family 2 member 6
SLC2A8	Solute carrier family 2 member 8
SLC2A12	Solute carrier family 2 member 12
GLUT13	_NA_
SLC2A14	Solute carrier family 2 member 14

EIF2AK4	Eukaryotic translation initiation factor 2 alpha kinase 4
EIF2S1	Eukaryotic translation initiation factor 2 subunit alpha
ATF4	Activating transcription factor 4
ALOX5	Arachidonate 5-lipoxygenase
ALOX12	Arachidonate 12-lipoxygenase, 12S type
ALOX15	Arachidonate 15-lipoxygenase
ALOX5	Arachidonate 5-lipoxygenase
ACSF2	Acyl-CoA synthetase family member 2
IREB2	Iron responsive element binding protein 2
GPX4	Glutathione peroxidase 4
HMGB1	High mobility group box 1
HMOX1	Heme oxygenase 1
NFE2L2	Nuclear factor, erythroid 2 like 2
ELAVL1	ELAV like RNA binding protein 1
SLC3A2	Solute carrier family 3 member 2
SLC7A11	Solute carrier family 7 member 11
TFAP2C	Transcription factor AP-2 gamma
SP1	Sp1 transcription factor
HBA1	Hemoglobin subunit alpha 1
NNMT	Nicotinamide N-methyltransferase

PLIN4	Perilipin 4
HIC1	HIC ZBTB transcriptional repressor 1
STMN1	Stathmin 1
RRM2	Ribonucleotide reductase regulatory subunit M2
CAPG	Capping actin protein, gelsolin like
HNF4A	Hepatocyte nuclear factor 4 alpha
NGB	Neuroglobin
YWHAE	Tyrosine 3-monooxygenase/tryptophan 5-monooxygenase activation protein epsilon
GABPB1	GA binding protein transcription factor subunit beta 1
AURKA	Aurora kinase A
MIR4715	microRNA 4715
RIPK1	Receptor interacting serine/threonine kinase 1
PRDX1	Peroxiredoxin 1
MIR30B	microRNA 30b

Supplementary Figure 2. Primer sequences of four ferroptosis-related lncRNAs for quantitative real-time PCR.

lncRNA	Forward primer	Backward primer
LNCSRLR	5′-CACTGTTCCAGGCACCAAGG-3′	5′-TGTCGCCAAAGAAGAGAACAGG-3′
